# Chronic ethanol administration exacerbates memory loss by altering N6-methyladenosine-mediated epigenetic signaling

**DOI:** 10.3389/fimmu.2025.1455994

**Published:** 2025-07-01

**Authors:** Yuanhang Liao, Fu Xu, Yuqing Yan, Sicheng Zhou, Na Liu, Baomin Dou, Nivetha Srinivasan, Weizheng Wang, Xiongwei Zhu, Jianghong Ye, Ying Xu

**Affiliations:** ^1^ Department of Anesthesiology, Rutgers University, the State University of New Jersey, Newark, NJ, United States; ^2^ Department of Pharmaceutical Sciences, School of Pharmacy, SUNY at Buffalo, Buffalo, NY, United States; ^3^ Division of Gastroenterology, Rutgers University, the State University of New Jersey, Newark, NJ, United States; ^4^ Department of Pathology, Case Western Reserve University, Cleveland, OH, United States

**Keywords:** MeRIP/RNA sequence, m 6 A modulation, epigenetics, immune cells infiltration, chronic intermittent ethanol

## Abstract

**Background:**

Chronic alcohol use disorder (AUD) is recognized as one of the most critical risk factors for the progression of Alzheimer’s disease (AD). Epigenetic and neuroimmune alterations are closely associated with the development of memory impairment related to AUD and AD.

**Methods:**

Adult APP/PS1 transgenic mice received intermittently intraperitoneal injections of ethanol (EtOH, 2.5 g/kg, i.p.) or vehicle with two “drug” treatment days, and one and two “drug-free” days every 7 days for 10 weeks. The novel object recognition (NOR) and Y-maze tests were performed to determine whether chronic ethanol treatment exacerbated memory impairment in these mice. The brain tissues were collected for pathological changes through MeRIP/RNA-sequence analyses and molecular biological assays.

**Results:**

The results suggested that chronic intermittent ethanol (CIE) treatment for 10 weeks exacerbated sporadic and spatial memory deficits in NOR and Y-maze tests in the APP/PS1 mice. The pathological assays revealed that CIE procedure increased Aβ plaque burden in the brain of the AD mice, which were consistent with memory behavioral deficits. The subsequent MeRIP/RNA sequence analyses showed that two genes, e.g. Rbm15b and Hnrnpa2b1, were related to N6-methyladenosine (m^6^A) methylation that plays an important role in the development of memory loss. These results were further supported by molecular biological and mRNA-microRNA-lncRNA ceRNA network analyses that demonstrated that the increased Rbm15b and decreased Hnrnpa2b1 were involved in synaptic dysfunction and neuroinflammation in CIE-induced memory impairment in these AD mice.

**Conclusions:**

The conclusion is drawn that m^6^A mediated epigenetic dysfunction and immune cells infiltration participate in chronic alcohol use disorder related memory loss in AD mice.

## 1 Introduction

Alzheimer’s disease (AD) is characterized by an irreversible cognitive and memory loss resulting from the oxidative stress-related amyloid-β (Aβ) aggregation and neuroinflammation (1). Chronic alcohol consumption is a critical risk factor in the development of AD pathology (2). This can lead to a series of symptoms, including progressive memory loss and psychiatric comorbidities such as depression and anxiety, all contributing to progressive neurodegeneration (3). Recent studies have demonstrated that feeding mice with ethanol-containing diets induces excessive Aβ expression, associated with increases in its precursor protein (APP) and the secretase enzymes in the brain responsible for the cleavage of APP to Aβ (2). Other studies suggest that alcohol abuse induced release of reactive oxidative species (ROS) and inflammatory cytokines contributes to the progression of neurodegeneration (4). These findings demonstrate that alcohol- and Alzheimer’s disease-related dementias (ADRD) may share a similar neurodegenerative signaling pathway highly related to oxidative stress and neuroinflammation (4–6).

Recent study has focused attention on the contributions of complex etiology, such as alcohol consumption, to ADRD through the influence of multiple susceptible gene regulatory networks (7). Epigenetic transcriptional regulation includes DNA methylation and demethylation, noncoding RNA regulation, and covalent histone modification through ATP-dependent chromatin remodeling (8, 9). Some studies demonstrate that abnormal epigenetic modifications may lead to disruptions in intracellular communication and potentially result in neurodegenerative outcomes, such as dementias associated with AD (10, 11). Indeed, epigenetic modulations are mainly attributable to ethanol metabolic stress by affecting oxidative metabolism of ethanol and methionine metabolism, which are implicated in the progression of neurodegeneration (12, 13). Analogous to DNA and histone modifications, recent studies demonstrated that RNA modifications could also lead to alterations in gene expression. N6-methyladenosine (m^6^A) is the most common internal modification in eukaryotic mRNAs (14) which is involved in the regulation of axon growth, synapse formation, and spine development. Aberrant m^6^A methylation has been found in AD patients and mouse models, as demonstrated by the downregulated mRNA levels of m^6^A methylation in the brains of mid-stage AD patients and AD mice (14, 15). This abnormal m^6^A methylation causes subsequent upregulation of inflammatory cytokines and neuronal loss, supporting the hypothesis that regulation of m^6^A methylation may play a crucial role in the development of ADRD (16). RNA modification induced gene alterations may allow extra regulation of gene-environmental interaction in alcohol abuse and its contribution to the risk for ADRD development (17) as increasing studies demonstrate that chronic ethanol consumption induces aberrant RNA methylation in the brain, thus increasing the risk of AUD development and memory impairment (18, 19).

Considering that m^6^A modification is dynamically regulated by methyltransferases, removed by demethylases, and recognized by m^6^A-binding proteins (20), the present study investigated whether and how chronic alcohol consumption exacerbate neurodegeneration triggered by epitranscriptomic changes in early-stage AD mouse models. This was conducted by analyzing alterations in epitranscriptomic modification and immune cell infiltration through behavioral, morphological, MeRIP/RNA-sequence, and molecular biological analyses. The results provide a new direction for potential therapeutics in the treatment of alcohol abuse-related ADRD.

## 2 Materials and methods

### 2.1 Animals

#### 2.1.1 Animals and housing

APP/PS1 mice at two months of age and age-matched wild-type (WT) C57 littermates, weighing 25–35 g, were purchased from Jackson Laboratory and bred in the animal center. Upon their arrival, the mice were accommodated five per cage under standard colony conditions, with a 12 -hour light/12-hour dark cycle (lights on at 7:00 a.m.). Experimental procedures were performed between 10:00-16:00. All experiments adhered to the guidelines outlined in the “NIH Guide for the Care and Use of Laboratory Animals” (revised 2014) and were approved by the Animal Care and Use Committee of Rutgers University, The State University of New Jersey.

#### 2.1.2 Chronic ethanol treatment procedure

To mimic human-like ethanol (EtOH) binge drinking in mice, the mice received intermittent daily intraperitoneal injections of EtOH (2.5 g/kg) or its vehicle (0.9% saline, Sal, i.p.). EtOH or vehicle was administered once daily for two consecutive days, followed by one and two “drug-free” days, repeated over a period of ten weeks. In brief, EtOH was administered on Monday and Tuesday, with Wednesday being EtOH-free. EtOH was administered again on Thursday and Friday with Saturday and Sunday being EtOH-free. This protocol was repeated for ten weeks. To prepare the EtOH for administration, a solution containing 20% alcohol (comprising anhydrous ethanol 20% and normal saline 80%) was utilized and administered to mice intraperitoneally (at a dose of 2.5g/kg). The alcohol dose was calculated using the formula: alcohol dose (μl) = body weight (g) × 15.8 (μl/g), with the coefficient derived from the alcohol content per gram (body weight) of mice.

Two cohorts of mice were divided into four groups, each cohort consisting of 5–7 mice/group (n=5-7): WT+vehicle (WT-veh), WT+EtOH (WT-e), APP/PS1+vehicle (APS-veh), and APP/PS1+EtOH (APS-e). Behavioral tests were conducted 2 hours after the last ethanol treatment. One cohort mouse was used for testing spatial memory in the Y-maze task; the other cohort were used for testing sporadic memory behavior in the novel object recognition test. The person who conducted the behavioral tests was blinded to the drug treatment and groups. After the behavioral tests, brains from one cohort of mice were used for MeRIP-sequence analysis. Half of the brains from the other cohort were used for molecular assays, such as immunoblot and PCR assays, while the other half were used for pathological assays, such as plaque analysis.

#### 2.1.3 Behavior tests

##### 2.1.3.1 Y-maze spontaneous alternation test

The behavioral tests were conducted blindly and administered to groups of mice. The Y-maze test was performed as previously described with minor modification (21). Each mouse underwent a 5-minute test session to explore the three arms of the Y-maze. The tracking system and analysis software from Noldus Information Technology (Virginia, USA) were used to analyze the entries into each arm. Spontaneous alternation (%) was examined as consecutive entries in three different arms, divided by the number of alternations, e.g., the total arm entries minus 2.

##### 2.1.3.2 Novel object recognition

The novel object recognition test was conducted as previously outlined with minor adjustments (22). The task comprised two phases: a training session (T1) on day 1 lasting 30 minutes and a test session (T2) on day 2 lasting 5 minutes. During the T1 session, the time spent exploring two identical objects was recorded as a1 and a2. In the T2 session, the time spent exploring the familiar and novel objects was recorded as “a” and “b”, respectively. The relative discrimination index d2 = (b-a)/(a+b). Actively touching or facing within 2 cm toward the object was chosen to quantify the time to explore the object by the tracking system and EthoVision XT17 software (Noldus Information Technology, Virginia, USA). Sitting on the object was not included in the calculations. The objects were wiped with 70% ethanol after each trial to prevent the olfactory cues.

After behavioral tests, mouse hippocampi were collected for MeRIP/RNA-sequence analyses, pathological assays, and molecular biological assays. Two mouse hippocampi were pooled together to create a single sample for subsequent sequence analyses and molecular biological assays.

#### 2.1.4 Thioflavin S staining

The hemibrain sections from mice per groups were mounted onto a glass slide and allowed to air dry completely for staining assay. The slide was immersed in 70% and 80% ethanol for 1 min and then incubated in thioflavin S solution (0.5% in 50% ethanol) for 15 min and washed by 80% ethanol and 70% ethanol for 1 min, respectively. After two times wash by distilled water, the slide was mounted with aqueous mounting media and allowed to dry. Then the coverslip was sealed with clear nail polish for visualizing the green fluorescence- stained plaque.

#### 2.1.5 Western blotting assay

The total proteins were extracted from brain tissues using RIPA lysis buffer (VWR, PA, USA). The lysates were then centrifuged at 12,000g for 30 min at 4 degrees Celsius, and the resulting supernatant was collected to determine protein concentration. Subsequently, 25 μg of protein was loaded into each lane and separated by 10% SDS-PAGE gels. The separated proteins were electro-transferred onto polyvinylidene difluoride (PVDF) membranes and then blocked in Tris-buffered saline with 0.1% Tween 20 (TBST) containing 5% BSA for 1 h at room temperature. The membranes were incubated with the following antibodies: anti-RNA binding motif protein 15 (Rbm15) (1:500, Cell Signaling Technology), anti-Rbm15b (1:500, Abcam), anti-Hnrnpa2b1 (1:500, Abcam), anti-Synaptophysin (1:500, Abcam), anti-PSD95 (1:500, Abcam), anti-β-actin (1:500, Abcam). The incubation was carried out on a rotator at 4 degrees Celsius overnight.

Following incubation, the membranes underwent three washes in TBST buffer, after which they were incubated with the anti-HRP-conjugated secondary antibody (1:5000, Abcam) for 1 hour at room temperature. The resulting antigen-antibody-peroxidase complexes were detected by adding the ECL kit (Thermo, Waltham, MA, USA) and visualized using the Bio-Rad Chemidoc Imaging System (Hercules, California, USA).

#### 2.1.6 Real-time quantitative RT-PCR

In the real-time quantitative RT-PCR assay, mRNA levels in the hippocampus were detected. RNA samples underwent reverse transcription using 6-nucleotide random primers and M-MLV reverse transcriptase (M0253, NEB). Quantitative PCR reactions were executed with SYBR™ Green Master Mix (43–856-12, Applied Biosystems) and specific primers on the StepOne 96-well Real-Time RT-PCR System (Applied Biosystems). The mRNA levels were normalized to the actin gene. Gene expression ratios were analyzed relative to the control set of the experiment.

The following primer pairs were used:

mouse β-actin, 5′-GTGACGTTGACATCCGTAAAGA3′ (sense)
and 5′- GTAACAGTCCGCCTAGAAGCAC -3′ (antisense);
mouse Rbm15, 5′-GTTCAAACGCTTCGGTGATGTA -3′ (sense)
and 5′-CACAAAGGCTACCCGCTCAT -3′ (antisense);
mouse Rbm15b, 5′- CACAGTGTTTCTGAGGTGGAGC -3′(sense)
and 5′- GCCTTGCCGTAGCCTATCTTT -3′(antisense);
mouse Hnrnpa2b1, 5′- CATTGATGGCAGGGTAGTTGAG − 3′ (sense)
and 5′- CCTTAATTCCACCAACAAACAGC − 3(antisense);
mouse Synaptophysin, 5′- ACCTCGGTGGTGTTTGGCTT − 3′ (sense)
and 5′-TGCCCGTAATCGGGTTGA − 3′ (antisense).
mouse PSD95, 5′- GCAGGTTGCAGATCGGAGAC − 3′ (sense)
and 5′- ACTGATCTCATTGTCCAGGTGCT− 3′ (antisense).

#### 2.1.7 ELISA assay

The levels of IL-1β (Tribioscience, USA), IL-10 (Tribioscience, USA), TNF-α (Tribioscience, USA), and CD-30 (Tribioscience, USA) were assessed based on the manufacturer’s instructions for the ELISA assay. Initially, standards and samples were dispensed into the ELISA plate and allowed to incubate at room temperature (RT) for 2 hours. After washing three times, the diluted Detection A was added to each well and incubated at RT for another 2 hours. The plate was then washed again. Subsequently, the diluted Detection B was added to each well and incubated at RT for 20 minutes. Following another round of washing, the Substrate Solution was added to each well and incubated for 10–20 minutes at RT. After adding the Stop Solution to each well and gently tapping the plate for mixing, the Optical Density (OD) value was determined at 450 nm. The results were calculated based on the standard curve.

### 2.2 Data analyses

#### 2.2.1 Statistical analysis for behavioral and morphological assays

The results were presented as mean ± SEM and analyzed using GraphPad Prism for behavioral and pathological testing. Behavioral and pathological differences among groups of vehicle-treated WT, ethanol-treated WT, vehicle-treated APP/PS1, and ethanol-treated APP/PS1 were analyzed using two-way ANOVA and t-test, respectively. A significance value of p<0.05 was established for the statistical tests.

#### 2.2.2 MeRIP/RNA-sequence and data pre-processing

##### 2.2.2.1 MeRIP-sequence analysis

Total RNAs were extracted, precipitated, and subjected to DNase I treatment to eliminate DNA contamination, followed by ethanol washing. The resulting product was dissolved in ultrapure water, and the RNA concentration was quantified. Subsequently, the RNA was randomly fragmented to approximately 200 nucleotides and then incubated with an anti-m^6^A antibody. The RNA reaction mixture was washed with immunoprecipitation (IP) buffer after immunoprecipitation. After extensive washing by low- and high-salt IP buffer at 4°C, the RNA reaction mixture was digested by Proteinase K followed by phenol-chloroform extraction and ethanol precipitation to elute m^6^A-enriched RNA fragments. Library construction was conducted on purified RNA using a kit following the manufacturer’s procedure (#56593, Cell Signaling, USA). The RNA sequence was determined using an Illumina Novaseq 6000 sequencing platform.

Low-quality bases were trimmed using trim Galore (version: 0.5.0). The filtering parameters applied to raw reads were: -phred 33, -stringency 2, -length 30, and -quality 20. Trim Galore was also used to remove adapter sequences, specifically Illumina True-seq adapters. And then the cleaned reads were aligned to the mm10 mouse genome using HISAT2 (version 2.1.0) with default parameters. The alignment file (SAM) was converted to a BAM file and underwent filtering following this procedure (1): duplicates were removed, retaining uniquely aligned reads (2); reads with low MAPQ (<30) were filtered out, while concordantly aligned read pairs were retained. With default parameters, the R package exomePeak (version 2.1.2) was employed to detect peaks and assess differential peaks from the filtered alignment file. Peaks exhibiting a p-value < 0.05 were regarded as significantly differential peaks.

##### 2.2.2.2 RNA-sequence analysis

Reference genome and gene model annotation files were downloaded from genome website (http://ftp.ensembl.org/pub/release-106/fasta/mus_musculus/dna/Mus_musculus.GRCm39.dna.toplevel.fa.gz & http://ftp.ensembl.org/pub/release-106/gtf/mus_musculus/Mus_musculus.GRCm39.106.gtf.gz). The index of the reference genome was constructed using Bowtie v2.0.6, and the paired-end clean reads were aligned to this reference genome using TopHat v2.0.9. Long noncoding RNAs (lncRNAs) were verified by four analyses, e.g., CPC, CNCI, CPAT, and pfam protein domain analyses. The index of the reference genome was built using Bowtie V2.0.6, and the paired-end clean reads were aligned to the reference genome using TopHat V2.0.9. Long noncoding RNAs (lncRNAs) were examined by four analyses, such as CPC, CNCI, CPAT, and pfam protein domain analyses. The analysis focused on the selection of the upstream 10,000 coding genes and downstream lncRNAs. The functions of these genes were analyzed as a predictor of the Cis target gene. The involvement of trans-target genes was identified through the interaction with lncRNAs and the respective expression levels. Pearson correlation coefficient analysis was applied to investigate the correlation between lncRNAs and protein-coding genes across different groups. To further elucidate potential trans-target genes, the blast software was utilized to verify the complementation relationship between lncRNA and mRNA bases.

##### 2.2.2.3 Gene set enrichment analysis

Gene sets were obtained from the MSigDB database which includes the Kyoto Encyclopedia of Genes and Genomes (KEGG), and Gene Ontology (GO). The genome annotation version GencodeV30 was employed to analyze the signaling and networks associated with the aforementioned genes. The analysis was conducted using the resources provided on https://www.gencodegenes.org/.

##### 2.2.2.4 Construction of the ceRNA network

The ceRNA network was identified through two primary steps. First, mRNAs were retrieved from the TargetScan database V8.0 following targeted miRNAs. Second, the NPInter database (Version 4.0) was utilized to predict the interaction between lncRNAs and miRNAs. And then the lncRNA-miRNA-mRNA ceRNA network was constructed. The interactions within the network were analyzed using the Cytoscape software, which is available at https://cytoscape.org/.

##### 2.2.2.4 Estimation of immune cell infiltration between different groups

To determine the proportions of immune cells in ethanol-treated AD mice, the CIBERSORT algorithm was used to quantify the relative infiltration of immune cells. The analyses compared the difference between 1) the vehicle-treated and ethanol-treated WT mice; 2) the vehicle-treated AD mice and vehicle-treated WT mice; and 3) vehicle-treated and ethanol-treated AD mice, respectively. The CIBERSORT algorithm encompassed molecular characterizations of 25 immune subsets.

## 3 Results

### 3.1 Chronic ethanol administration exacerbates cognitive deficits in novel object recognition and Y-maze tests in early stage of AD mice

**Figure 1:**
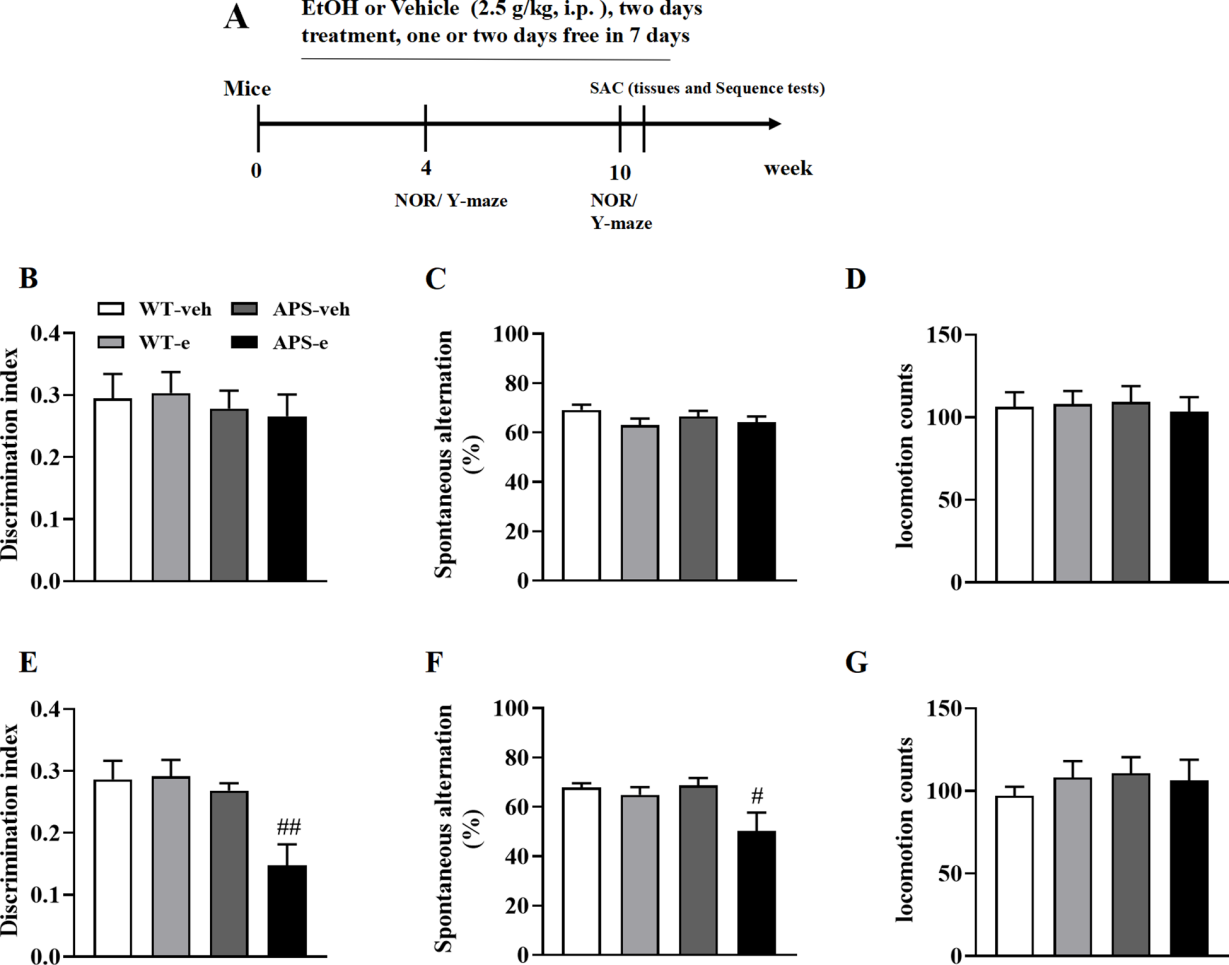
Chronic ethanol treatment paradigm **(A)** for 4- **(B-D)** or 10-week **(E-G)** induced cognitive impairment in novel object recognition **(B, E)** and Y maze **(C, F)** tests in mice. Two months of age APP/PS1 mice and the age-matched wild-type (WT) littermates were treated with vehicle or 20% ethanol (2.5 g/kg, i.p.) for 4- or 10-week. Locomotion counts were determined for 10 min when mice were treated with ethanol for 4- or 10-week **(D, G)**. Behavioral tests were conducted 2 h after last ethanol treatment. All values are expressed as mean ± SEM. ^#^p < 0.05, ^##^p < 0.01 vs. vehicle-treated APP/PS1 mice.

The initial indication of memory loss in patients with AD is episodic and sporadic memory deficits. This cognitive decline is mirrored by a reduction in the discrimination index in the novel object recognition test in mice (23). In the present study, two-month-old WT and APP/PS1 mice were treated ethanol (2.5 g/kg) by intraperitoneal injection (i.p.) starting at two months of age for 4 and 10 weeks following a regimen of two consecutive days of treatment and one or two drug free days (**Figure 1A**). Treatment of ethanol for 4 weeks did not induce significant episodic and sporadic memory deficits in either WT or AD mice, as no significant difference in the discrimination index (DI) was observed between these two groups (**Figure 1B**). However, the DI in AD mice treated with ethanol for 10 weeks was significantly reduced (**Figure 1E**, p<0.01). Further study revealed that the locomotion counts remained unchanged in these mice receiving ethanol for either 4 or 10 weeks (**Figures 1D, G**).

The Y-maze task was used to determine spatial memory difference following ethanol treatment for 4 or 10 weeks beginning at 2 months of age. WT and APP/PS1 mice treated with ethanol for 4 weeks showed a trend toward decreased cognitive abilities, as evidenced by a reduced tendency toward spontaneous alternation (**Figure 1C**). This cognitive impairment was exacerbated when these mice received ethanol for 10 weeks, as shown by a significant reduction in spontaneous alternations when APS mice were compared to their vehicle-treated littermates (**Figure 1F**, p<0.05).

### 3.2 Chronic ethanol administration increases Aβ plaques in early stage of AD mice

**Figure 2:**
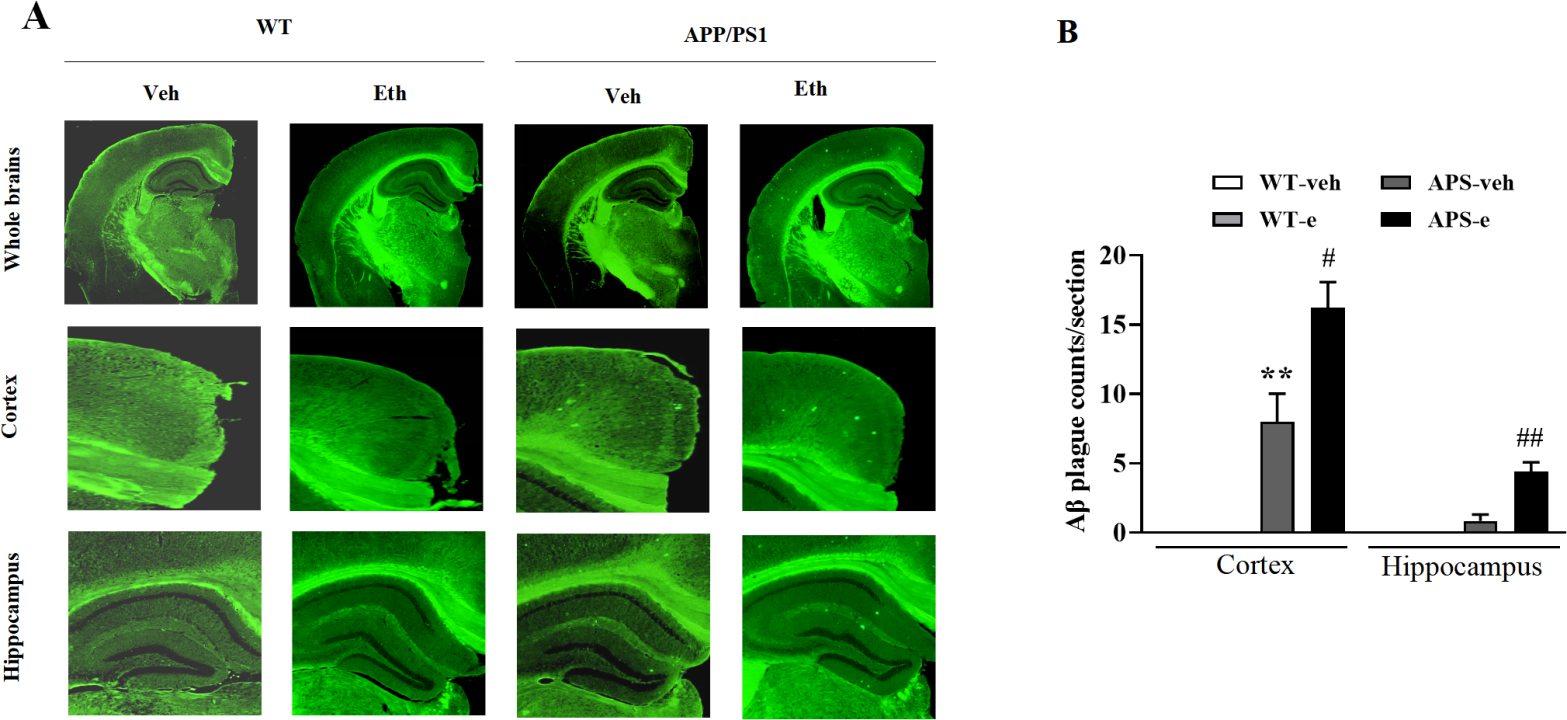
Ethanol-treated APP/PS1 mice increased Aβ plaques. Two months of age APP/PS1 mice and the age-matched wild-type (WT) littermates were treated with vehicle or 20% ethanol (2.5 g/kg, i.p.) for 10 weeks. Aβ plaque burden was detected by Thioflavin S staining **(A, B)**. Values are expressed as mean ± SEM. ^**^ p < 0.01 vs. vehicle-treated WT mice. ^#^ p < 0.05, ^##^ p <0.01 vs. vehicle-treated APP/PS1 mice.

The aggregation of Aβ into plaques within the AD brain is a fundamental characteristic of AD pathology. This process is related to the disruption of neural communication that plays a crucial role in memory loss associated with AD. To examine the effects of ethanol on Aβ deposits, the fluorescent dye staining assay was used to detect plaques in the brain of mice that received treatment for 10 weeks. As shown in **Figure 2**, no plaque accumulation in the hippocampus and cortex was found in the WT mice with or without alcohol treatment. A few sporadic amyloid plaques were observed in the cortex and the hippocampus in vehicle-treated APP/PS1 mice. However, pathological evaluation revealed the presence of amyloid plaques throughout the brain of APP/PS1 mice receiving ethanol for 10 weeks. The total numbers of plaques were significantly increased in the hippocampus and cortex compared to those of vehicle-treated APP/PS1 littermates (**Figures 2A, B**, p<0.05; p<0.01).

### 3.3 The m^6^A-related regulators mediate chronic ethanol consumption induced cognitive impairment in early stage of AD mice

**Figure 3:**
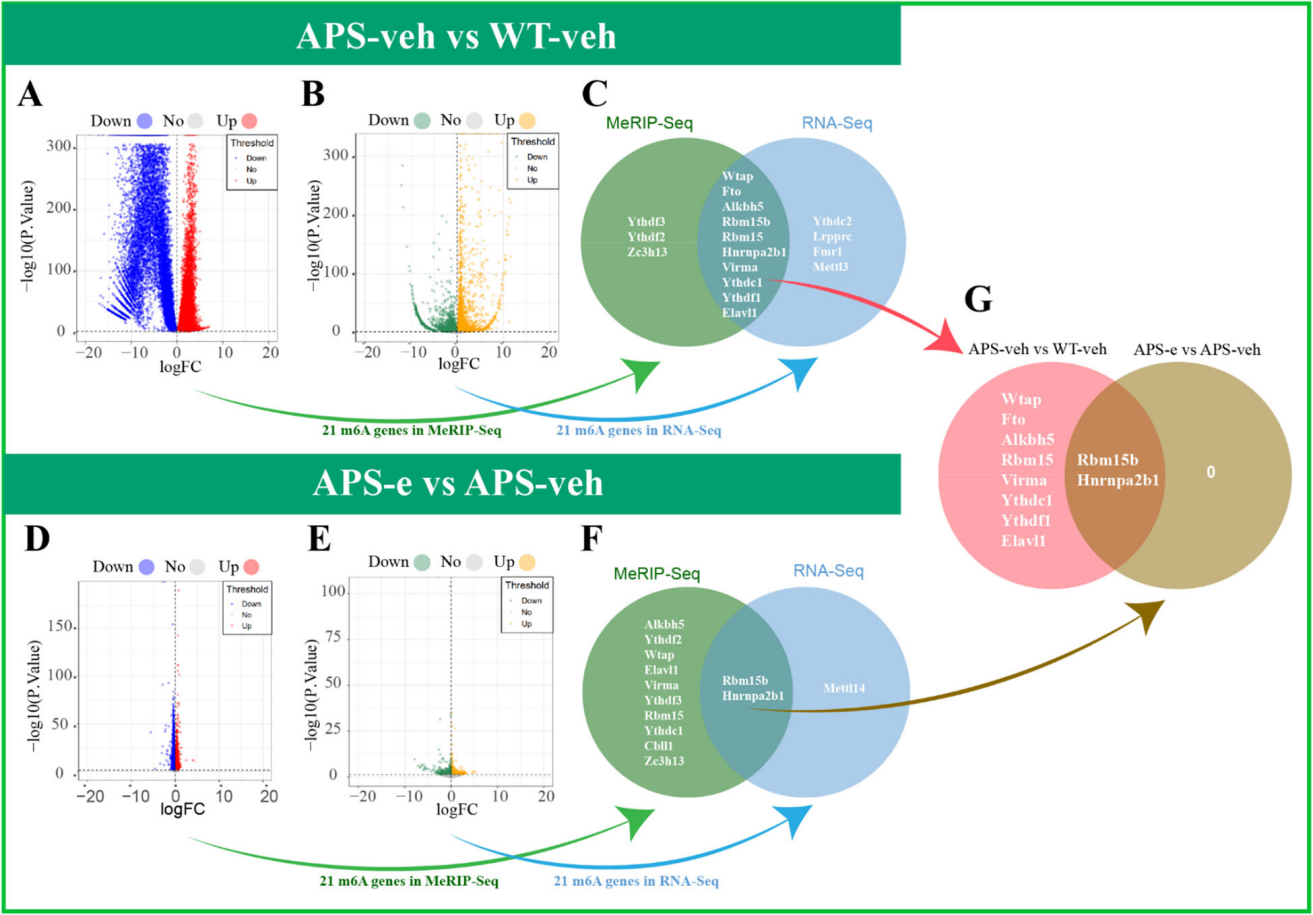
Differential expression analysis of m^6^A-related genes were conducted in the hippocampus of AD mice treated with ethanol for 10 weeks. **(A)** The MeRIP-Seq volcano plot showed that 12517 m^6^A peaks (corresponding to 5698 genes) were up-regulated and 12957 m^6^A peaks (corresponding to 4213 genes) were down-regulated in APP/PS1 mice. Each red dot showed an upregulated m^6^A peaks, and each blue dot showed a downregulated m^6^A peaks. **(B)** The RNA-Seq volcano plot showed that 3995 genes were up-regulated and 4747 genes were down-regulated in APP/PS1 mice. Each orange dot showed an up-regulated genes, and each green dot showed a down-regulated genes. **(C)** The Venn diagram revealed that 10 m^6^A-related genes overlapped between differentially expressed genes from m^6^A-MeRIP-seq and RNA-seq analyses. The green plot showed m^6^A-MeRIP-seq differentially expressed genes. The blue plot showed RNA-seq differentially expressed genes. **(D)** The MeRIP-Seq volcano plot showed that 6074 m^6^A peaks (corresponding to 2289 genes) were up-regulated and 6383 m^6^A peaks (corresponding to 2167 genes) were down-regulated in ethanol-treated APP/PS1 mice. Each red dot showed an upregulated m^6^A peaks, and each blue dot showed a downregulated m^6^A peaks. **(E)** The RNA-Seq volcano plot showed that 691 genes were up-regulated, and 712 genes were down-regulated in ethanol-treated AD mice. Each orange dot showed an up-regulated genes, and each green dot showed downregulated genes. **(F)** The Venn plot showed that two m^6^A-related genes were in the intersection of m^6^A-MeRIP-seq differentially expressed genes and RNA-seq differentially expressed genes. The green plot showed m^6^A-MeRIP-seq differentially expressed genes. The blue plot showed RNA-seq differentially expressed genes. **(G)** The Venn plot showed that two m^6^A-related genes were in the intersection of subgroups (APS-veh vs WT-veh) differentially expressed genes and subgroups (APS-e vs APS-veh) differentially expressed genes. The pink plot showed subgroups (APS-veh vs WT-veh) differentially expressed genes. The brown plot showed subgroups (APS-e vs APS-veh) differentially expressed genes.

To explore the mechanism underlying chronic ethanol consumption-induced cognitive impairment and progressive neurodegeneration in early-stage AD mice, two types of sequencing analyses, i.e. Methylated RNA immunoprecipitation (MeRIP) and RNA sequencing analyses, were conducted. As shown in **Figure 3A**, MeRIP sequencing analysis revealed differential regulation of various m^6^A-modified genes, with a total of 12,517 upregulated m^6^A peaks (corresponding to 5698 genes) and 12,957 downregulated m^6^A peaks (corresponding to 4213 genes) totally, in comparison of vehicle-treated AD mice to vehicle-treated WT littermates. Among these changed genes, two were m^6^A erasers, including Alkbh5 and Fto; six were readers, such as Ythdf1, Ythdf2, Ythdf3, Hnrnpa2b1, Ythdc1, and Elavl1; and five were writers, including Zc3h13, Wtap, Rbm15, Rbm15b, and Virma. The subsequent RNA sequencing analyses showed 3995 up-regulated and 4747 down-regulated genes in the hippocampus of AD mice as compared to those of age-matched WT littermates (**Figure 3B**). Further mRNA expression analysis suggested that these genes were regulated by fourteen m^6^A genes, which were consistent with the results of MeRIP analysis. As shown in **Figure 3C**, a Venn diagram of ten various m^6^A regulator genes in AD mice revealed an overlap between the results of MeRIP and RNA sequencing analyses. When these AD mice were treated with ethanol for 10 weeks, 6,383 m^6^A peaks (corresponding to 2167 genes) were down-regulated, and 6,074 m^6^A peaks (corresponding to 2289 genes) were up-regulated in the hippocampus compared to those of vehicle-treated AD mice (**Figure 3D**). Further study showed changes in mRNA levels, with 712 down-regulated and 691 up-regulated genes’ change observed (**Figure 3E**). In **Figure 3F**, two m^6^A regulator genes, Rbm15b (writer) and Hnrnpa2b1 (reader), exhibited overlapping changes in both MeRIP-seq and RNA-seq analyses. This convergence indicates a potential role for these m^6^A regulators in mediating the observed alterations in RNA methylation patterns in alcohol-related neurodegeneration (**Figure 3G**). The results of the differential expression analysis of m^6^A-related genes between the WT-veh and WT-e groups are presented in **Supplementary Figure 1**. These findings provide insight into the impact of ethanol treatment on m^6^A-related gene expression in wild-type mice.

### 3.4 The validation of m^6^A-related regulators expression in the hippocampus of early stage of AD

**Figure 4:**
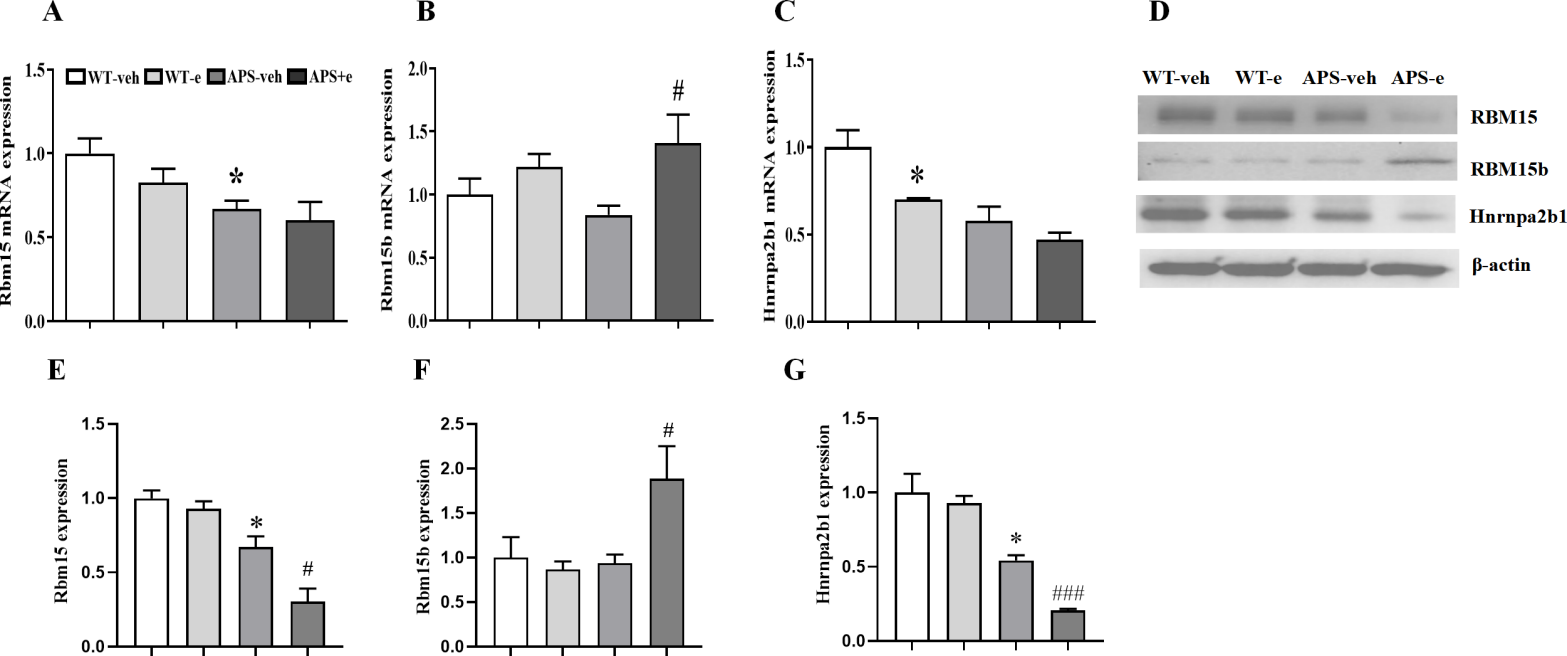
Chronic ethanol treatment for 10 weeks altered m^6^A related genes expression in AD mice. Relative mRNA expression of Rbm15 **(A)**, Rbm15b **(B)** and Hnrnpa2b1 **(C)** compared to vehicle-treated WT mice. Western blotting **(D)** detected the expression of Rbm15 **(E)**, Rbm15b **(F)** and Hnrnpa2b1 **(G)**. Values are expressed as mean ± SEM. *p < 0.05 vs.vehicle-treated WT mice. ^#^ p < 0.05, ^###^ p < 0.001 vs. vehicle-treated APP/PS1 mice.

Since Rbm15b is a paralogue of Rbm15, the mRNA and protein levels of Rbm15 were examined. As shown in **Figure 4A**, Rbm15 mRNA expression in the hippocampus of AD mice was significantly decreased compared to that of WT mice (p<0.05). A subsequent study showed that Rbm15b mRNA level was increased (**Figure 4B**, p< 0.05), while Hnrnpa2b1 mRNA expression had tendency to decrease in the ethanol-treated AD mice (**Figure 4C**).

In the immunoblot assay, Rbm15 protein levels were decreased in AD mice compared to WT littermates, with a further decrease observed in ethanol-treated AD mice (**Figures 4D, E**, p< 0.05; p< 0.05). However, Rbm15b protein expression was significantly increased in ethanol-treated AD mice, whereas Hnrnpa2b1 protein levels were significantly decreased (**Figures 4D, F, G**, p< 0.05; p< 0.001), indicating that ethanol treatment induces differential changes of these m^6^A-regulator genes in AD mice.

### 3.5 The m^6^A-related regulators are regulated by ceRNA network in the early stage of AD

**Figure 5:**
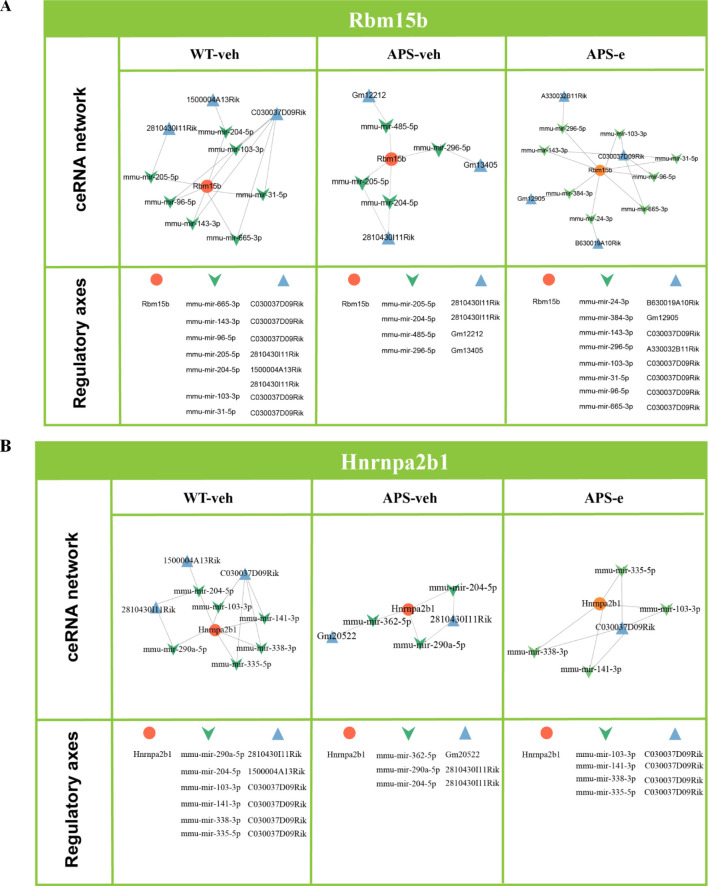
ceRNA analyses revealed that Rbm15b and Hnrnpa2b1 related signaling pathways **(A, B)** were influenced in APP/PS1 mice treated with ethanol for 10 weeks.

To explain how upstream ceRNA networks regulate m^6^A-modified genes, two m^6^A-related regulators, particularly Rbm15b and Hnrnpa2b1, and the upstream ceRNA axes were analyzed. **Figures 5A** and 5B illustrated that the ceRNA regulatory axes for Rbm15b and Hnrnpa2b1 differed in ethanol-treated AD mice. Considering that the ceRNA network includes both microRNA- and lncRNA-related pathways, the present study analyzed how microRNA- and lncRNA-related pathways regulate Rbm15b- and Hnrnpa2b1-dependent signaling. As shown in **Figure 5A**, the Rbm15b gene was regulated by eight ceRNA regulatory axes in the WT group, while four ceRNA regulatory axes were detected in the APP/PS1 group. Additionally, eight ceRNA regulatory axes were related to ethanol treatment. However, two lncRNAs were different when comparing AD mice to their relative WT littermates, e.g., GM12212 and Gm13405. After treatment with ethanol for 10 weeks in AD mice, eight lncRNAs were observed differently than vehicle-treated AD littermates. In **Figure 5B**, the ceRNA network for Hnrnpa2b1 showed that six, three, and four ceRNA regulatory axes were related to WT, AD, and ethanol-treated AD groups, respectively. However, 8 lncRNAs were utterly different in ethanol-treated AD mice compared to vehicle-treated littermates.

### 3.6 Chronic ethanol consumption exacerbates the progression of neurodegeneration through m^6^A regulators related post-synaptic signaling

**Figure 6:**
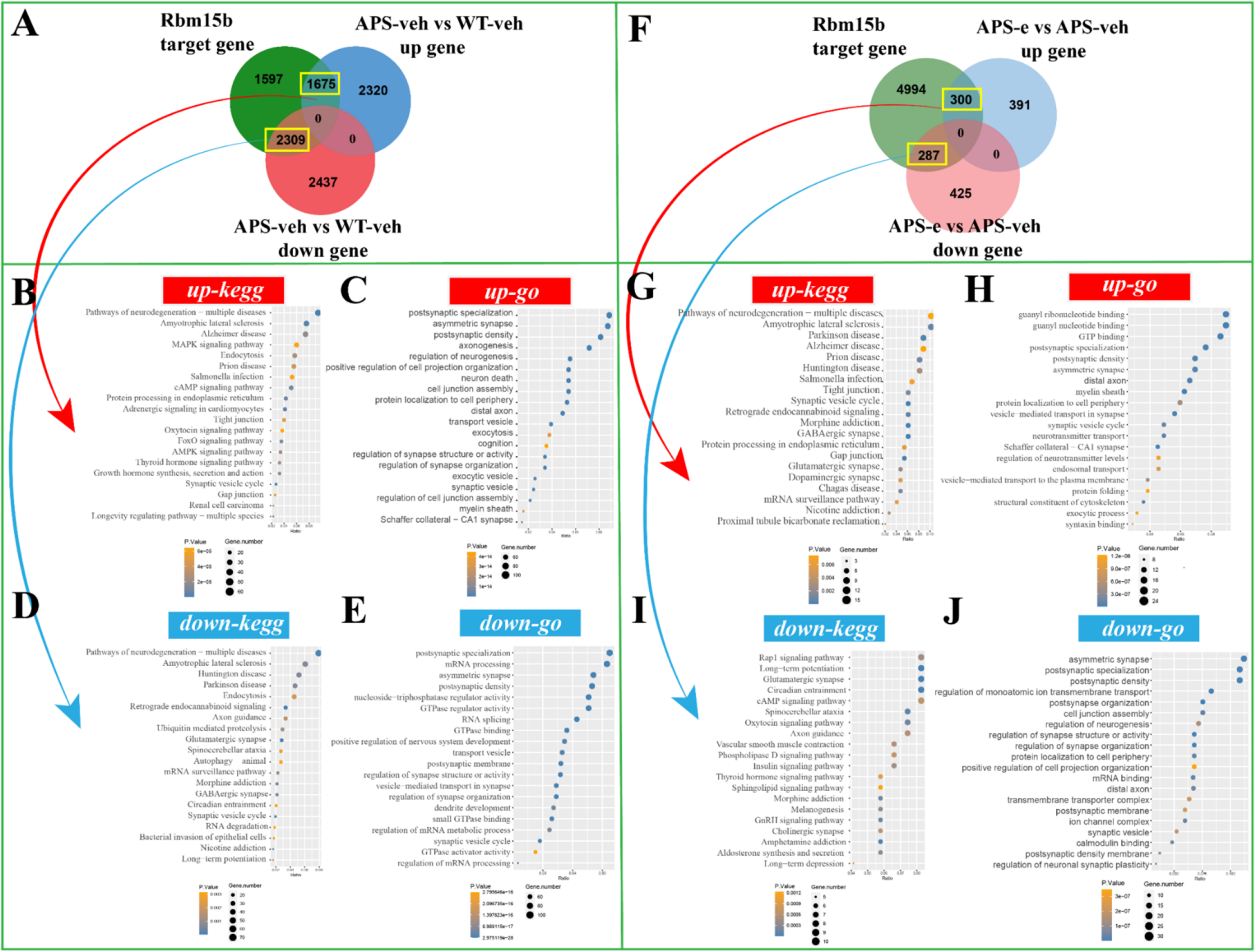
The enrichment analysis of Rbm15b target gene was conducted in AD mice treated with ethanol for 10 weeks. **(A)** The Venn diagram revealed that 1675 up-regulated genes and 2309 down-regulated genes were in the intersection of Rbm15b target genes in AD group. **(B, C)** The KEGG and GO enrichment analyses were conducted on the up-regulated target genes of Rbm15b. **(D, E)** The KEGG and GO enrichment analyses were conducted on the down-regulated target genes of Rbm15b. **(F)** The Venn diagram revealed that 300 up-regulated genes and 287 down-regulated genes were in the intersection of Rbm15b target genes in ethanol-treated AD group. **(G, H)** The KEGG and GO enrichment analyses were conducted on the up-regulated target genes of Rbm15b. **(I, J)** The KEGG and GO enrichment analyses were conducted on the down-regulated target genes of Rbm15b.

**Figure 7:**
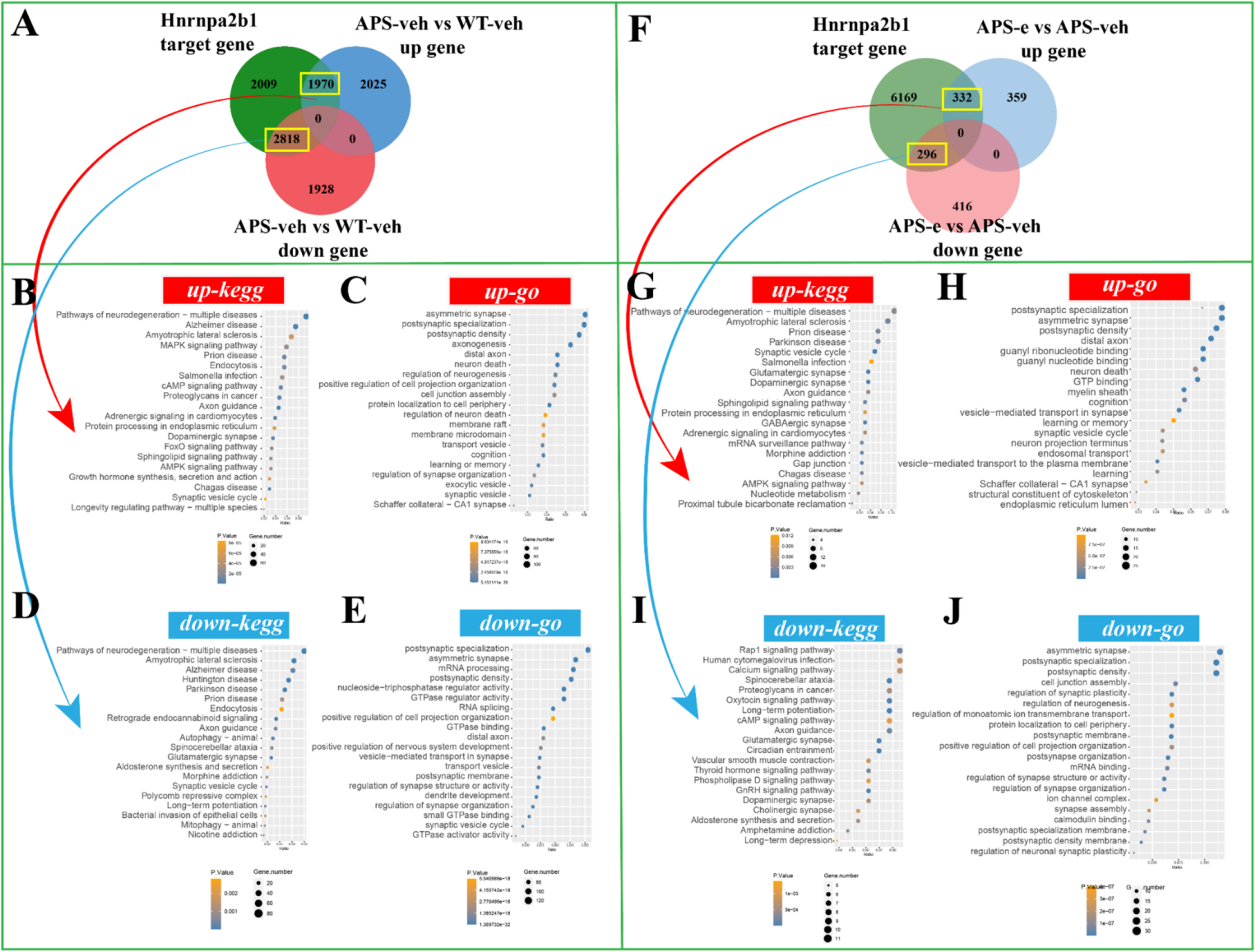
The enrichment analysis of Hnrnpa2b1 target gene was conducted in AD mice treated with ethanol for 10 weeks. **(A)** The Venn diagram revealed that 1970 up-regulated genes and 2818 down-regulated genes were in the intersection of Hnrnpa2b1 target genes in AD group. **(B, C)** The KEGG and GO enrichment analyses were performed on the up-regulated target genes of Hnrnpa2b1. **(D, E)** The KEGG and GO enrichment analyses were performed on the down-regulated target genes of Hnrnpa2b1. **(F)** The Venn diagram revealed that 332 up-regulated genes and 296 down-regulated genes were in the intersection of Hnrnpa2b1 target genes in ethanol-treated AD group. **(G, H)** The KEGG and GO enrichment analyses were performed on the up-regulated target genes of Hnrnpa2b1. **(I, J)** The KEGG and GO enrichment analyses were perform on the down-regulated target genes of Hnrnpa2b1.

To determine the downstream effects of the target genes of Rbm15b and Hnrnpa2b1 in KEGG and GO analyses, the correlations of Rbm15b and Hnrnpa2b1 with cognitive deficits and neurodegeneration were further investigated through RNA sequencing assays. As shown in **Figures 6A**, there were 1675 up-regulated genes in the APP/PS1 group compared to WT littermates, which correlated with Rbm15b target genes. These up-regulated genes were mainly associated with pathways of neurodegeneration, particularly in neurodegeneration and cAMP signaling pathways as revealed by KEGG analysis (**Figure 6B**), and the postsynaptic specialization and plasticity as verified by GO enrichment analysis (**Figure 6C**). We also found that 2,309 genes related to Rbm15b target genes were downregulated in AD mice (**Figure 6A**). These down-regulated genes were primarily involved in pathways of neurodegeneration, glutamatergic synapse signaling, mRNA processing, and postsynaptic density, based on KEGG and GO analyses (**Figures 6D, E**). Further study showed that 300 up-regulated genes changed in ethanol-treated APP/PS1 mice, which correlated with the target genes of Rbm15b (**Figure 6F**). The up-regulated genes were primarily related to neurodegeneration and GABAergic synapse as evidenced by KEGG analysis (**Figure 6G**). These genes were also involved in asymmetric synaptic and GTP binding, as shown in GO enrichment analysis (**Figure 6H**). In addition, a total of 287 down-regulated genes were found in ethanol-treated APP/PS1 mice, which were correlated with the target genes of Rbm15b, as shown in **Figure 6F**. These down-regulated genes were primarily associated with the glutamatergic synapse and cAMP signaling pathways, as verified by the KEGG analysis (**Figure 6I**), and were involved in post-synapse organization and regulation of synapse structure and activity through the GO enrichment analysis (**Figure 6J**). In the subsequent study, there were a total of 1,970 up-regulated genes closely related to Hnrnpa2b1 target genes in APP/PS1 mice when compared to WT littermates. These genes were mainly involved in pathways of neurodegeneration, development of Alzheimer’s disease, MAPK and cAMP signaling, and axon-genesis, as shown in KEGG and GO analyses (**Figures 7A–C**). Moreover, 2,818 down-regulated genes in different APP/PS1 group were correlated with Hnrnpa2b1 target genes. These down-regulated genes also were primarily involved in pathways of neurodegeneration, development of Alzheimer’s disease, glutamatergic synapse signaling, and postsynaptic- and neuronal-plasticity associated pathways, as shown in KEGG and GO analyses (**Figures 7D, E**). The subsequent study revealed that the target genes of Hnrnpa2b1 were correlated with a total of 332 up-regulated genes in the ethanol-treated APP/PS1 group (**Figure 7F**). All these genes were primarily involved in pathways of neurodegeneration, glutamatergic synapse, and postsynaptic modification as indicated in KEGG and GO analyses (**Figures 7G, H**). Moreover, Hnrnpa2b1 target genes were related to 296 down-regulated different genes in the ethanol-treated APP/PS1 mice, which were mainly associated with pathways of calcium metabolic, cAMP and glutamatergic signaling, postsynaptic specialization, and density modulation, as shown in the KEGG and GO analyses (**Figures 7I, J**). The results of the Virma and Ythdc1 target gene enrichment analyses between WT-veh and WT-e groups were shown in **Supplementary Figure S2**.

### 3.7 Chronic ethanol consumption is linked to post-synaptic modulation in the progression of AD pathology

**Figure 8:**
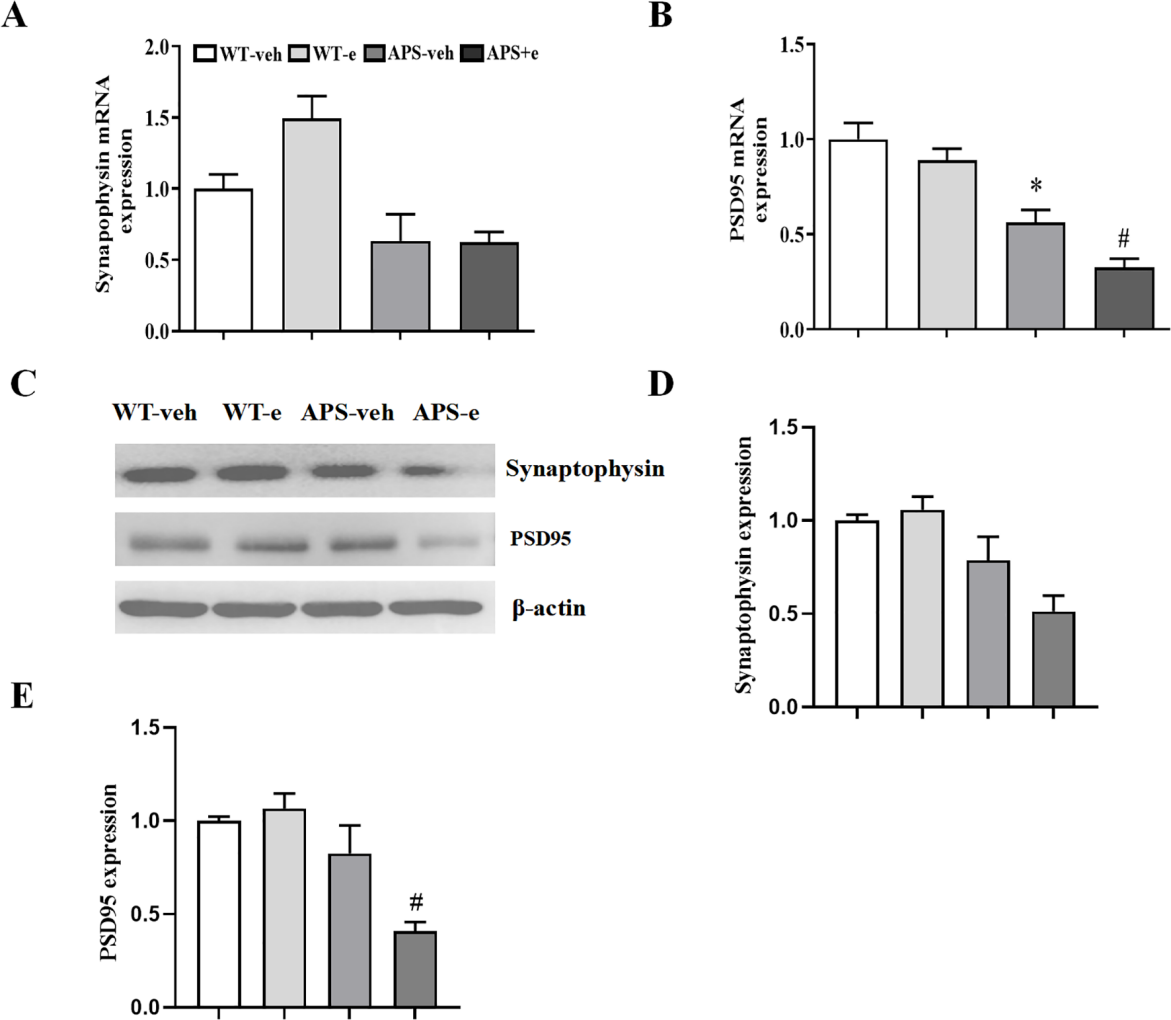
Chronic ethanol treatment for 10 weeks altered synaptic related genes expression in AD mice. Reverse transcription-quantitative PCR detected the expression of Synaptophysin **(A)** and PSD95 **(B)**. Western blotting **(C)** showed the expression of Synaptophysin **(D)** and PSD95 **(E)**. All values are expressed as mean ± SEM. * p < 0.05 vs. vehicle-treated WT mice. ^#^ p < 0.05 vs. vehicle-treated APP/PS1 mice.

Synaptic dysfunction participates in neuronal atrophy and the deterioration of cognitive impairment. Real-time RT-PCR assay showed that the mRNA levels of post-synaptic proteins PSD-95 were decreased in both vehicle-treated and ethanol-treated APP/PS1 mice (**Figure 8B**, p < 0.05; p < 0.05). However, the mRNA level of the presynaptic protein synaptophysin did not show significant changes in ethanol-treated AD mice (**Figure 8A**, p > 0.05). It is noted that ethanol-treated AD mice significantly decreased the protein levels of post-synaptic protein, e.g., PSD-95 (**Figures 8C, E**, p < 0.05). While the presynaptic protein, e.g. synaptophysin, only had tendency to decrease after treatment with ethanol for 10 weeks in APP/PS1 mice (**Figures 8C, D**). This finding suggests chronic ethanol consumption plays a crucial role in post-synaptic modulation at the transcriptional and translational level.

These results demonstrate that chronic ethanol consumption may participate in the translation process of synaptic regulation. Both genes were also validated for their mRNA expression using qRT-PCR (data not shown). The results exhibited a consistent trend with the findings of the mRNA sequencing analysis, thereby corroborating the reliability of the mRNA sequencing results.

### 3.8 Immune cells infiltration and neuroinflammation are related to chronic ethanol consumption related AD pathology

**Figure 9:**
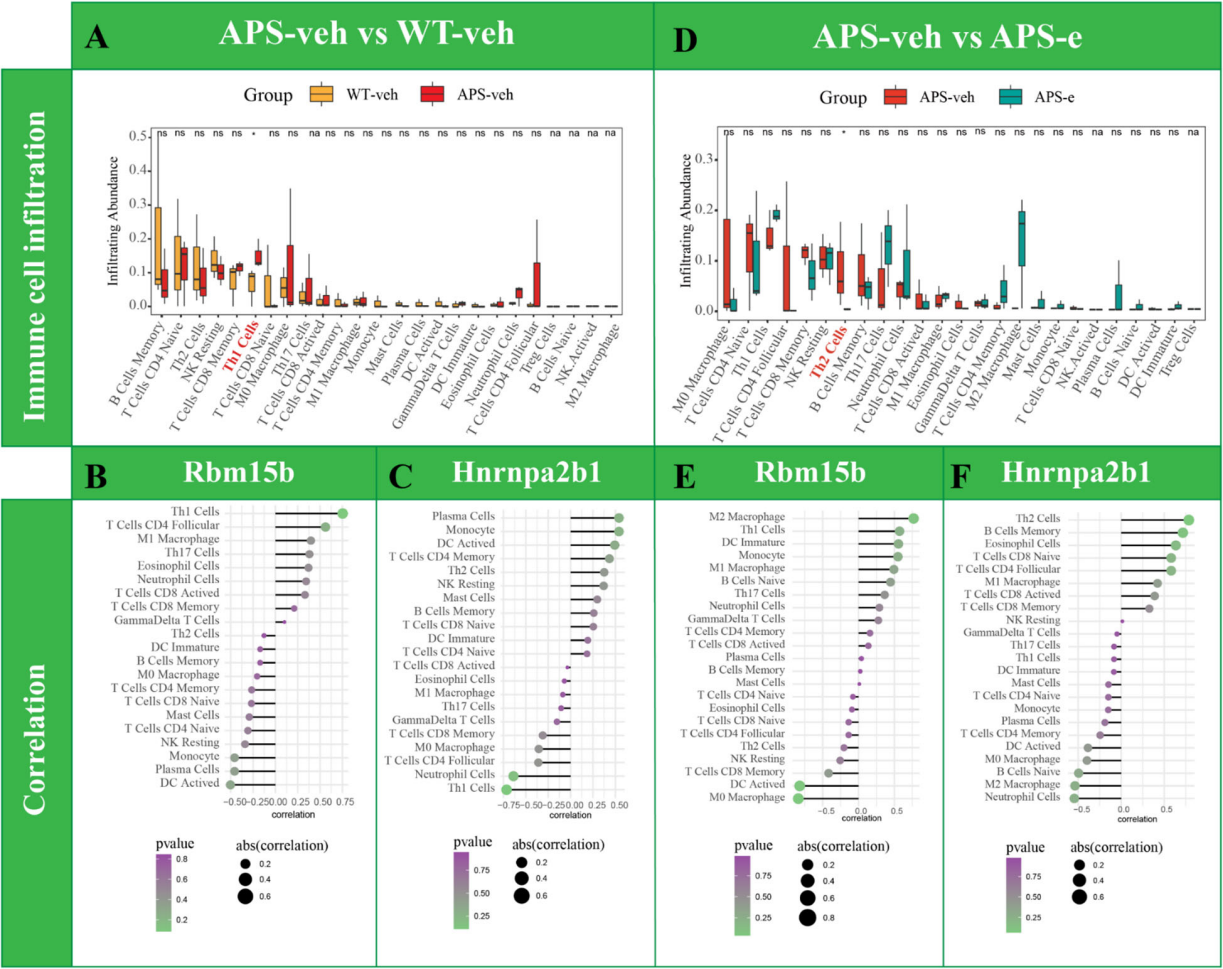
Analysis of immune cell infiltration change was conducted in AD mice treated with ethanol. Analysis of immune cell infiltration was conducted in WT-veh and APS-veh groups **(A)**. Lollipopplot of the correlation analysis was performed between Rbm15b expression and immune cell infiltration in AD mice **(B)**. Lollipopplot of the correlation analysis was performed between Hnrnpa2b1 expression and immune cell infiltration in AD mice **(C)**. Analysis of immune cell infiltration was performed in ethanol-treated AD mice **(D)**. **(E)** Lollipop plot of the correlation analysis was performed between Rbm15b expression and immune cell infiltration in ethanol-treated AD mice. **(F)** Lollipop plot of the correlation analysis was performed between Hnrnpa2b1 expression and immune cell infiltration in ethanol-treated AD mice.

The CIBERSORT algorithm analysis was used to quantify the relative infiltration of immune cells in APP/PS1 mice that received ethanol for 10 weeks. As shown in **Figure 9A**, Th1 cells were increased significantly in APP/PS1 mice (p< 0.05), indicating the immune cells participating in AD pathology. As shown in **Figures 9B, C**, the correlation of Rbm15b and Hnrnpa2b1 with 25 immune cells was further analyzed in AD mice. The Rbm15b gene was positively correlated with Th1 cell levels, while the Hnrnpa2b1 gene was negatively correlated with Th1 cell expression. Further analysis found that the infiltration abundance of Th2 cells was decreased in ethanol-treated AD mice (**Figure 9D**, p< 0.05). However, the infiltration abundance of B cells memory, M0 macrophage, and T cells CD4 follicular showed a tendency to change in ethanol-treated AD mice, but the difference was not significant. Moreover, the Rbm15b gene was found to correlate with Th2 levels negatively. In contrast, the Hnrnpa2b1 gene was positively correlated with Th2 levels (**Figures 9E, F**), further supporting that chronic ethanol consumption significantly exacerbates AD pathology by modulation of immune responses and inflammation. The immune cell infiltration analysis results of m^6^A-related genes among WT-veh and WT-e groups are shown in **Supplementary Figure S3**.

### 3.9 Chronic ethanol administration increases pro-inflammatory cytokines and decreases anti-inflammatory markers in early stage of AD mice

**Figure 10:**
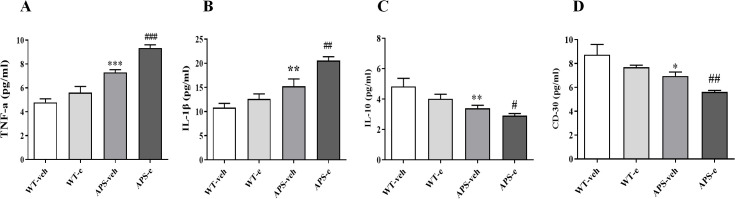
Chronic ethanol treatment for 10 weeks increased pro-inflammatory markers TNF-α **(A)** and IL-1β **(B)** and decreased anti-inflammatory markers IL-10 **(C)** and CD 30 **(D)** in AD mice. Values are expressed as mean ± SEM. * p < 0.05, ** p <0.01, *** p < 0 .001 vs. vehicle-treated WT mice. ^#^ p < 0.05, ^##^ p < 0.01, ^###^ p < 0.001 vs. vehicle-treated APP/PS1 mice.

**Figure 11:**
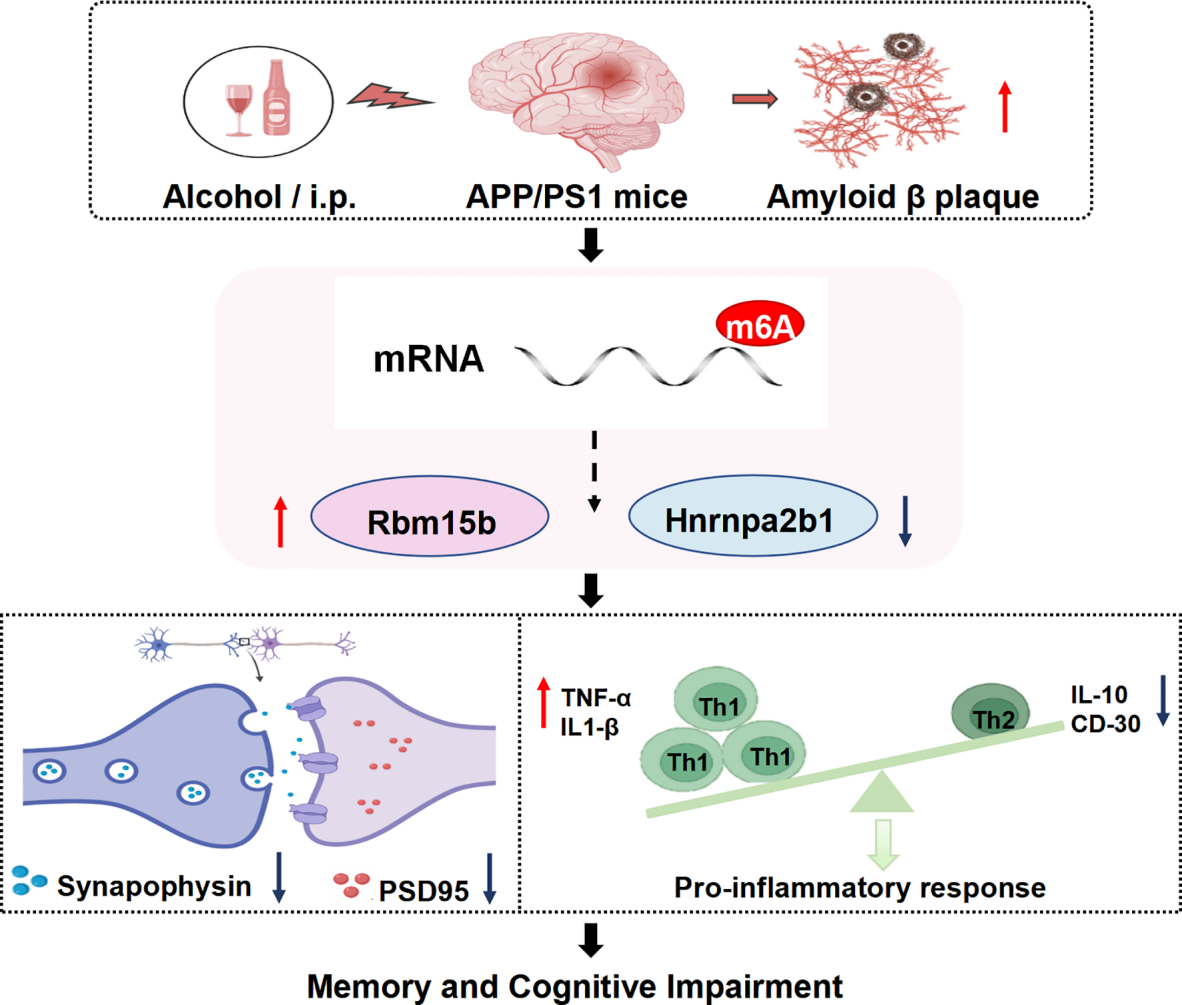
Chronic intermittent ethanol treatment for 10 weeks induces memory loss and increases in plaque burdens in APP/PS1 mice. Subsequent MeRIP/RNA sequence analysis showed that chronic ethanol treatment caused changes in mRNAs and protein levels of N6 methyl-adenosine (m6A), i.e. an increase in Rbm15b level and a decrease in Hnrnpa2b1 level. These changes resulted in dysfunction in synaptic protein expression and stimulated Th1 and Th2 cells, causing the infiltration of immune factors. The pathological changes contribute to the progression of AD, resulting in the deterioration of episodic memory (a hallmark symptom of early AD) and spatial memory (a characteristic symptom of late AD).

To determine whether neuroinflammation is involved in ethanol-related AD pathology, we analyzed the pro-inflammatory cytokines, including Tumor Necrosis Factor-α (TNF-α) and Interleukin-1β (IL-1β), as well as anti-inflammatory markers such as Interleukin-10 (IL-10) and CD30 in the hippocampus of ethanol-treated AD mice. The ELISA assay showed that the levels of TNF-α and IL-1β significantly increased in the hippocampus of both vehicle-treated APP/PS1 mice and ethanol-treated APP/PS1 mice, as compared to their respective control groups (**Figures 10A, B**, *p*< 0.001; *p*< 0.01). As shown in **Figure 10C, D**, the IL-10 and CD30 levels were significantly decreased in the hippocampus of vehicle-treated APP/PS1 mice and ethanol-treated APP/PS1 mice, respectively (*p*< 0.01 & *p*< 0.05; *p*< 0.05 & *p*< 0.01). These findings suggest that ethanol consumption aggravates the inflammatory response in brain regions related to memory deficits, which was summarized in **Figure 11**.

## 4 Discussion

The present study found that episodic memory (a major symptom of the early stage of AD) and spatial memory (a late-stage symptom of AD) were getting worse after treatment of adult AD mice with ethanol for 10 weeks. The pathological assay suggested that ethanol administration increased plaque burden in the brain of AD mice, particularly in the cortex and hippocampus. This increase may be related to cytokine release in these brain regions. The subsequent MeRIP/RNA-sequence analyses suggested that chronic ethanol treatment-induced cognitive impairment was related to the dysfunction of the mRNA-microRNA-lncRNA ceRNA network and post-synaptic remodeling. Of particular interest were the findings that two mRNAs, e.g., Rbm15b and Hnrnpa2b1, were related to the stimulation of Th1 and Th2 cells and the subsequent infiltration of immune cells. This further supports the hypothesis that chronic ethanol consumption causes dysfunction in Rbm15b and Hnrnpa2b1-dependent signaling pathways, which correlates with stimulating immune cell infiltration and cytokine expression. These processes could contribute to the destruction of synaptic remodeling and exacerbate the progression of AD.

Alcohol use disorder is characterized by a chronic relapsing brain disease with an abnormal emotional state such as depression, anxiety, and chronic alcohol abuse. This disorder may also be responsible for progressive neurocognitive deficits and severe dementia due to the structural or functional dysregulation of blood-brain barrier (BBB) or neurodegeneration (24, 25). A combined effects of ethanol toxicity and nutritional deficiencies could result in gradual memory deterioration and long-term neurological impairment (26). Alcohol abuse is ranked as the second leading, with a 40-60% incidence among AD patients in the US (27). However, the etiological factors contributing to the development of cognitive impairment such as alcohol abuse and its susceptibility to Aβ aggregation, require further clarification. Some studies suggest that alcohol use disorder induces temporary cognitive deficits, neuropsychiatric and neurodegenerative symptoms (4). These effects may be attributed to an excessive oxidative stress-related neuroimmune response and cell injury (28). The present study established a pathological connection between chronic alcohol use and the development of memory and cognitive deficits in AD mice, examined from an epigenetic perspective. Generally, exploring novel objects in the NOR test reflects the implicit form of sporadic or episodic memory, indicating the “what” aspect of the learning and memory process. Similarly, the results of Y-maze reflect intact spatial memory function by an increase in spontaneous alternation, which provide insight into the “where” aspect of memory processing. The present study showed that the administration of ethanol to WT mice for 10 weeks induced a tendency to decrease the discrimination index and spontaneous alternations in the NOR and Y-maze tests. However, cognitive and memory performance became worsen in ethanol-treated AD mice, demonstrating that chronic ethanol treatment exacerbated the development of AD pathology. These results are consistent with some studies that demonstrate that excessive alcohol drinking during adulthood exacerbates the trajectory of the abnormal aging process, leading to the development of AD-related dementias (29, 30). However, other studies argue that ethanol exposure could significantly extend the lifespan of Caenorhabditis elegans, resulting in the hypothesis that moderate ethanol consumption may benefit humans (31). This hypothesis is not currently widely accepted with more studies providing contrary evidence of chronic ethanol’s harmful effects (32). Recent attention focuses on whether and how excessive alcohol exposure exacerbates Aβ42 induced toxicities (33, 34). The present study demonstrated that relatively long-term heavy drinking exacerbated cognitive impairment during adulthood, as evidenced by that ethanol treatment for 10 weeks increased the Aβ plaque burden in the brain of 4.5-month-old AD mice, particularly in the cortex and hippocampus. These findings further demonstrate that excessive alcohol exposure can worsen the neurodegeneration.

Epigenetic dysfunction induced alterations of gene expression in the brain is recognized as one of the critical pathophysiological changes of aging-related neurodegeneration (9, 35). m^6^A RNA methylation and demethylation are the most prevalent internal RNA modifications in eukaryotes, regulating various aspects of RNA metabolism and its upstream and downstream signaling (36). However, whether m^6^A modification is perturbed in ethanol-induced learning and memory impairment associated with AD remains unclear. Previous studies demonstrated that m^6^A modification was installed by m^6^A methyltransferases (METTL3/14, WTAP, RBM15/15B, and KIAA1429, collectively termed ‘writers’), recognized by m^6^A binding proteins (YTHDF1/2/3, IGF2BP1 and HNRNPA2B1, also named as “readers”) and reversed by demethylases (FTO and ALKBH5, collectively termed ‘erasers’) (37). Recent study demonstrated that Mettl3-mediated m^6^A modification plays a crucial role in metabolism disorders such as alcohol use disorder (38). However, the changes in Mettl3 transcription levels in the chronic ethanol treated AD mice were not significant in the present study (data not shown). The possibility may be that Mettl3 expression is influenced by the different duration and doses of ethanol administration. In the present study, m^6^A and transcriptome sequence analyses showed that treatment of ethanol for 10 weeks resulted in two genes change in the brain, e.g., Rbm15b and Hnrnpa2b1. These two genes were closely correlated with pathways that regulate the postsynaptic specialization and primary immunodeficiency. We noticed that silencing of Rbm15 may inhibit tumor cell viability and suppress tumor growth in the xenograft mouse model (39). As a paralogue of RBM15, Rbm15b binds to the m^6^A-methylation complex and recruits it to specific site in RNA, acting as a repressor in several signaling pathways (40). Resent study suggested that the hub Rbm15b regulator targets are enriched in immune cells differentiation and glutamatergic synapse (41). Further analysis supports a close relationship between this m6A modification-related gene, e.g. Rbm15b, and immunological and synaptic dysfunction. Hnrnpa2b1 complexes with heterogeneous nuclear RNA influence pre-mRNA processing, mRNA metabolism, and transport, contributing to the oligomerization of tau protein and neuronal tangle formation (42). Neurons regulate the proteostasis process at synapses during oxidative stress, which plays a crucial role in cell-cell communication during memory formation. Recent study found that a dominant mutation of Hnrnpa2b1 impaired stress-mediated localization of some RNA-binding proteins in maintaining proteostasis in dendrites of cultured motor neurons, indicating its regulation of synaptic function (43). However, whether and how these two genes regulate the neurodegenerative process are not yet clarified. We found that Rbm15 expression was downregulated in AD mice treated with ethanol, while Rbm15b was significantly upregulated in these mice. On the contrary, ethanol treatment significantly reduced Hnrnpa2b1 expression in AD mice. These findings suggest that Rbm15, particularly Rbm15b, and Hnrnpa2b1, play critical roles in neurodegeneration during the progression of chronic alcohol consumption induced memory loss, particularly AD pathology.

A growing body of evidence indicates that long non-coding RNAs (lncRNAs) can act as competitive endogenous RNAs (ceRNAs) by binding to microRNAs (miRNAs), thereby influencing the expression of target genes (44–46). The present study demonstrated that the ceRNA regulatory axes for Rbm15b and Hnrnpa2b1 differed in ethanol-treated AD mice, indicating that these two genes are involved in chronic alcohol administration via upstream ceRNA regulations. Further analyses of ceRNA networks found that microRNA- and lncRNA-related pathways regulate alcohol-induced Rbm15b and Hnrnpa2b1-dependent signaling. Comparing the different m^6^A lncRNA-miRNA-mRNA ceRNA regulatory networks, we found that alcohol may influence the ceRNA regulatory network through m^6^A methylation and demethylation, promoting neuroinflammation and AD development. The KEGG and GO analyses showed that Rbm15b and Hnrna2b1 were closely correlated with 25 immune cells and genes that could regulate neuroinflammation. The further ELISA assay showed that the neuroinflammation-related genes such as TNF-a and IL-1β were significantly increased in ethanol-treated AD mice. In contrast, the anti-inflammatory genes, such as IL-10 and CD30, were significantly decreased in these mice, demonstrating that ethanol-induced cognitive impairment is related to immune deficiency and inflammatory response. These findings were consistent with the previous studies, which demonstrated that Rbm15b and Hnrnpa2b1 are oxidative stress-related protein kinases that may be responsible for an increased risk of neurodegenerative processing (47, 48). Considering that synaptic dysfunction contributes to neuronal loss and the deterioration of memory and cognition (49), the present study also examined the expression of pre-synaptic protein synaptophysin and post-synaptic protein -95 (PSD-95) in the hippocampus of ethanol-treated AD mice. The results showed that chronic ethanol administration significantly decreased both mRNA and protein levels of PSD-95 expression. The KEGG and GO analyses further support the correlations of Rbm15b and Hnrnpa2b1 with post-synaptic modulation in the development of AD related dementias.

Collectively, the present study suggested that long-term consumption of alcohol exacerbates memory and cognitive deficits by alteration of epigenetic m^6^A modulation and stimulation of proinflammation infiltration via mRNA-microRNA-lncRNA ceRNA network, which likely contributes to neurodegenerative pathology in the early stage of AD.

## Data Availability

The datasets presented in this study can be found in online repositories. The names of the repository/repositories and accession number(s) can be found in the article/**Supplementary Material**.

## Supplementary material

Supplementary Figure 1 The differential expression analysis of m^6^A-related genes in the hippocampus of mice was performed between wild-type mice treated with vehicle or ethanol for 10 weeks. **(A)** The MeRIP-Seq volcano plot showed that 12989 m^6^A peaks (corresponding to 4274 genes) were up-regulated, and 12441 m^6^A peaks (corresponding to 5757 genes) were down-regulated in the WT-e group compared to the WT-veh group. Each red dot showed an upregulated m^6^A peaks, and each blue dot shows a downregulated m^6^A peaks. **(B)** The RNA-Seq volcano plot showed that 5514 genes were up-regulated, and 4377 genes were down-regulated in the WT-e group compared to WT-veh group. Each orange dot showed an up-regulated genes, and each green dot showed a down-regulated genes. **(C)** The Venn plot revealed that 11 m^6^A-related genes were in the intersection of differentially expressed genes from m^6^A-MeRIP-seq and RNA-seq analyses. The green plot showed differentially expressed genes from m^6^A-MeRIP-seq analysis. The blue plot showed differentially expressed genes from RNA-seq analysis.

Supplementary Figure 2 The enrichment analysis of Virma and Ythdc1 target genes in the hippocampus of mice was conducted. The comparation was from wild-type mice treated with ethanol for 10 weeks and the respective vehicle treated group. **(A)** The Venn plot revealed that 2,602 genes were in the intersection of Virma target genes and up-regulated expressed genes in the subgroups (WT-e vs WT-veh). Additionally, the Venn plot showed that 111 genes were in the intersection of Virma target genes and down-regulated expressed genes in the subgroups (WT-e vs WT-veh). **(B, C)** The results of KEGG and GO enrichment analyses of up-regulated Virma target genes. **(D, E)** Results of KEGG and GO enrichment analyses of down-regulated Virma target genes. **(F)** The Venn plot showed that 3226 genes were in the intersection of Ythdc1 target genes and up-regulated expressed genes in the subgroups (WT-e vs WT-veh). Additionally, the Venn plot also showed that 2235 genes were in the intersection of Ythdc1 target genes and down-regulated expressed genes in the subgroups (WT-e vs WT-veh). **(G, H)** Results of KEGG and GO enrichment analyses of up-regulated Ythdc1 target genes. **(I, J)** Results of KEGG and GO enrichment analysis of down-regulated Ythdc1 target genes.

Supplementary Figure 3 The immune cell infiltration analysis of m^6^A-related genes was conducted among different subgroups in the hippocampus of WT mice treated with ethanol for 10 weeks. **(A)** The results of immune cell infiltration analysis in the subgroups (WT-veh and WT-e group). **(B)** The lollipop plot of the correlation between Virma expression and immune infiltrating cells in the subgroups (WT-veh and WT-e). **(C)** The lollipop plot of the correlation between Ythdc1 expression and immune infiltrating cells in the subgroups (WT-veh and WT-e).
