# Supplementary figures and images for: Chronic ethanol administration exacerbates memory loss by altering N6-methyladenosine-mediated epigenetic signaling
Source: Front Immunol. 2025 Jul 1;16:1455994. doi: 10.3389/fimmu.2025.1455994 (PMC12259454; doi:10.3389/fimmu.2025.1455994)

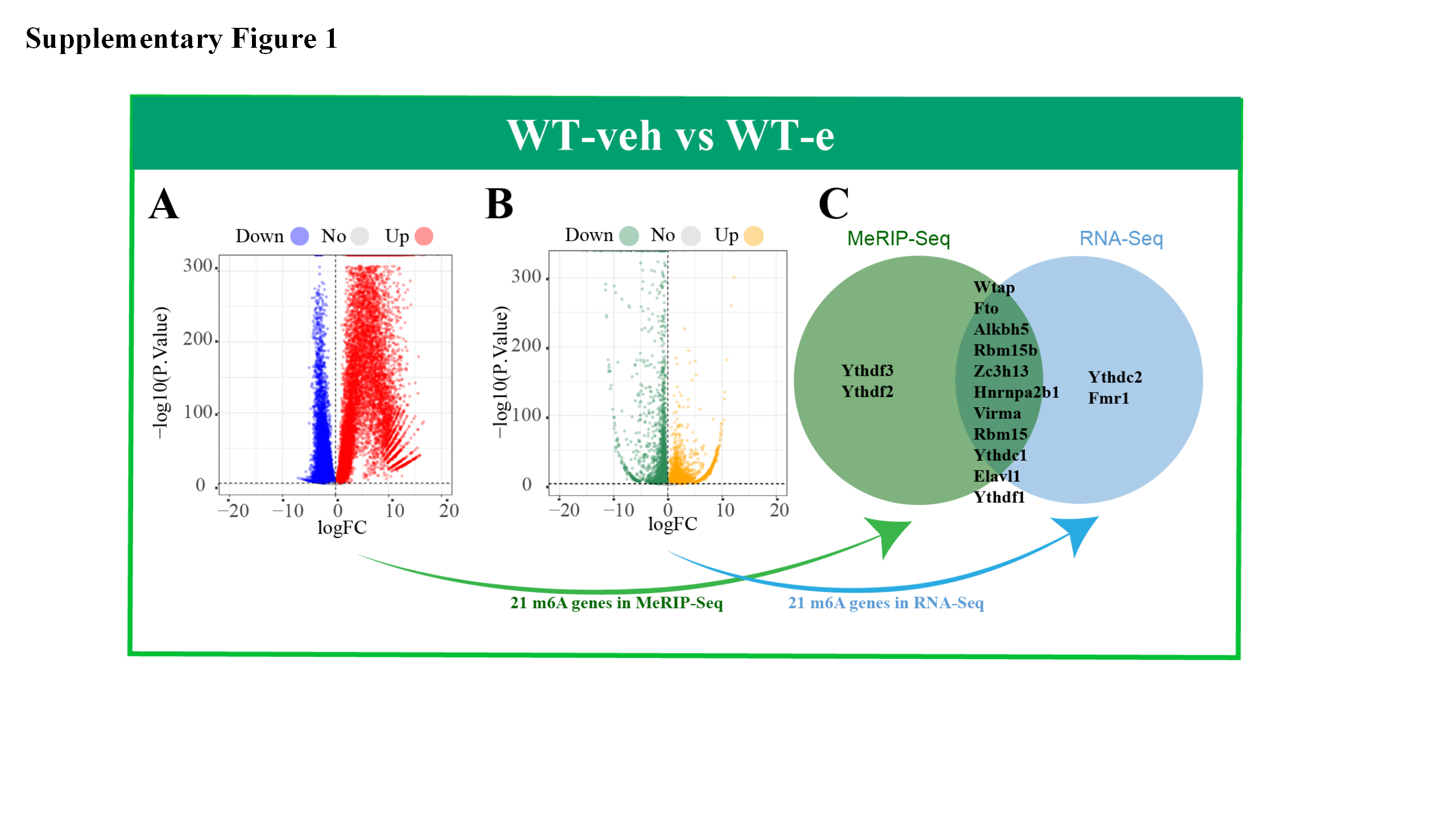

Supplement: Supplementary Figure 1 — The differential expression analysis of m6A-related genes in the hippocampus of mice was performed between wild-type mice treated with vehicle or ethanol for 10 weeks. (A) The MeRIP-Seq volcano plot showed that 12989 m6A peaks (corresponding to 4274 genes) were up-regulated, and 12441 m6A peaks (corresponding to 5757 genes) were down-regulated in the WT-e group compared to the WT-veh group. Each red dot showed an upregulated m6A peaks, and each blue dot shows a downregulated m6A peaks. (B) The RNA-Seq volcano plot showed that 5514 genes were up-regulated, and 4377 genes were down-regulated in the WT-e group compared to WT-veh group. Each orange dot showed an up-regulated genes, and each green dot showed a down-regulated genes. (C) The Venn plot revealed that 11 m6A-related genes were in the intersection of differentially expressed genes from m6A-MeRIP-seq and RNA-seq analyses. The green plot showed differentially expressed genes from m6A-MeRIP-seq analysis. The blue plot showed differentially expressed genes from RNA-seq analysis. [file Image1.tif]

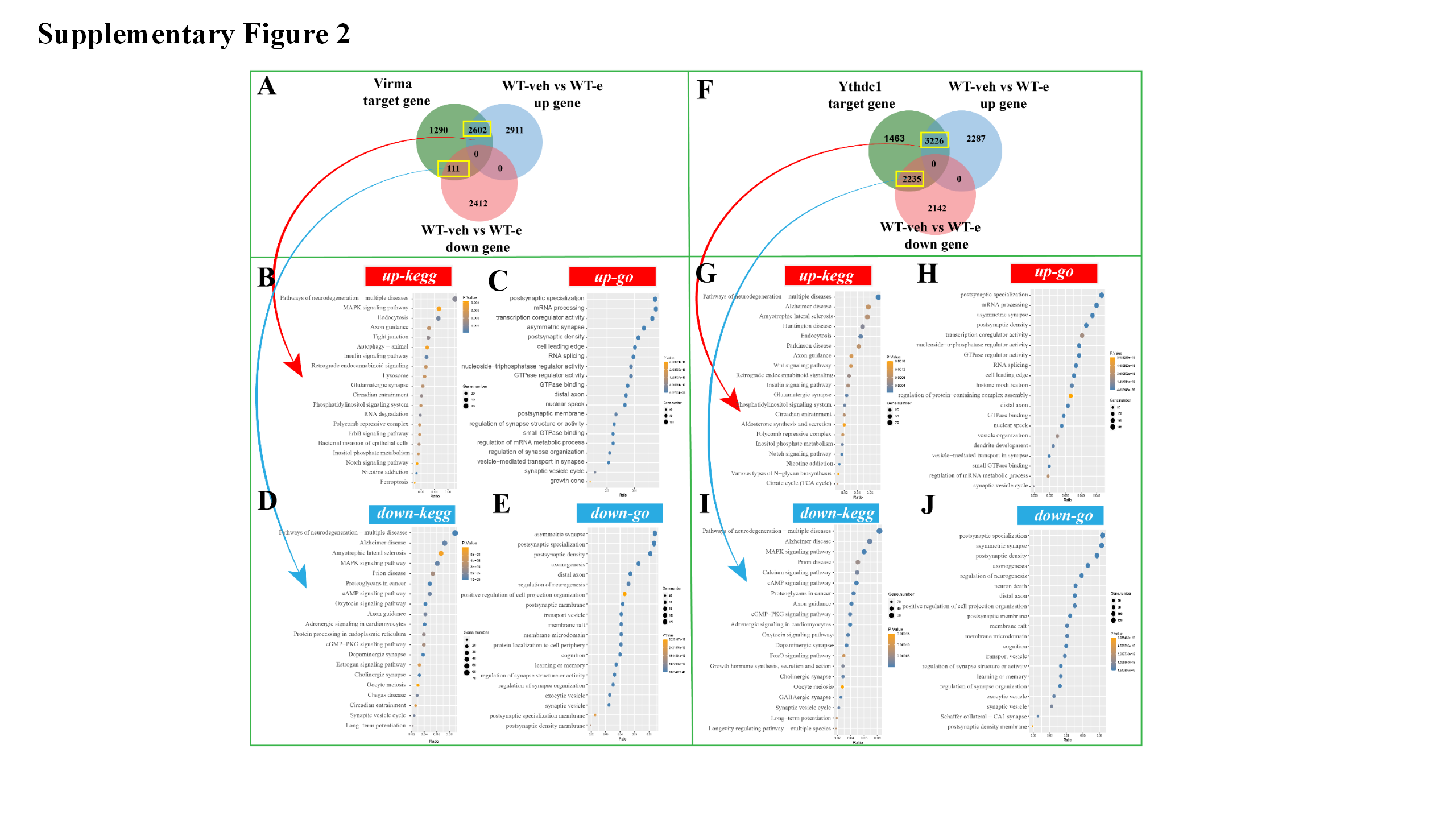

Supplement: Supplementary Figure 2 — The enrichment analysis of Virma and Ythdc1 target genes in the hippocampus of mice was conducted. The comparation was from wild-type mice treated with ethanol for 10 weeks and the respective vehicle treated group. (A) The Venn plot revealed that 2,602 genes were in the intersection of Virma target genes and up-regulated expressed genes in the subgroups (WT-e vs WT-veh). Additionally, the Venn plot showed that 111 genes were in the intersection of Virma target genes and down-regulated expressed genes in the subgroups (WT-e vs WT-veh). (B, C) The results of KEGG and GO enrichment analyses of up-regulated Virma target genes. (D, E) Results of KEGG and GO enrichment analyses of down-regulated Virma target genes. (F) The Venn plot showed that 3226 genes were in the intersection of Ythdc1 target genes and up-regulated expressed genes in the subgroups (WT-e vs WT-veh). Additionally, the Venn plot also showed that 2235 genes were in the intersection of Ythdc1 target genes and down-regulated expressed genes in the subgroups (WT-e vs WT-veh). (G, H) Results of KEGG and GO enrichment analyses of up-regulated Ythdc1 target genes. (I, J) Results of KEGG and GO enrichment analysis of down-regulated Ythdc1 target genes. [file Image2.tif]

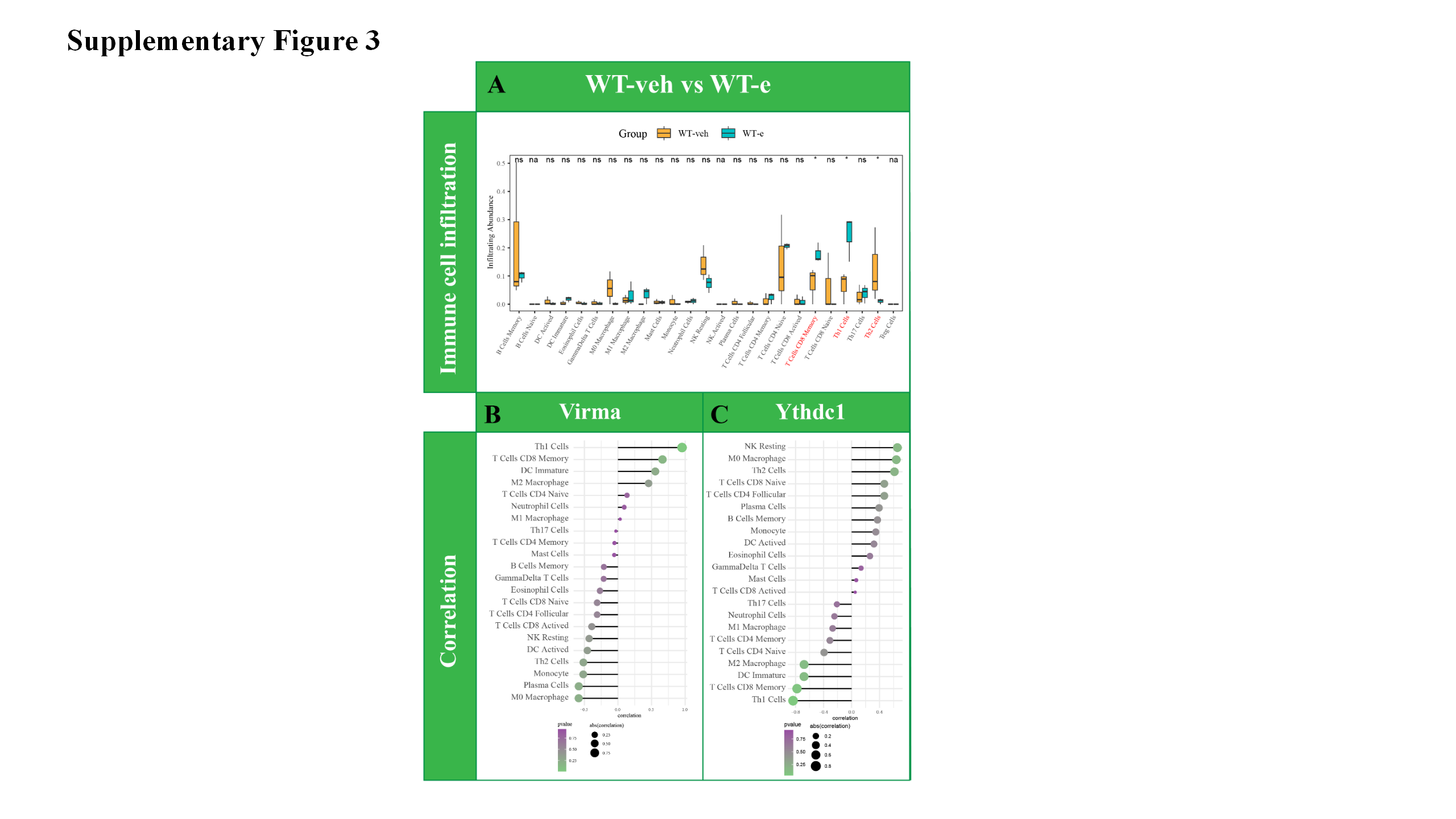

Supplement: Supplementary Figure 3 — The immune cell infiltration analysis of m6A-related genes was conducted among different subgroups in the hippocampus of WT mice treated with ethanol for 10 weeks. (A) The results of immune cell infiltration analysis in the subgroups (WT-veh and WT-e group). (B) The lollipop plot of the correlation between Virma expression and immune infiltrating cells in the subgroups (WT-veh and WT-e). (C) The lollipop plot of the correlation between Ythdc1 expression and immune infiltrating cells in the subgroups (WT-veh and WT-e). [file Image3.tif]
